# The osteoarthritis bone score (OABS): a new histological scoring system for the characterisation of bone marrow lesions in osteoarthritis

**DOI:** 10.1016/j.joca.2022.01.008

**Published:** 2022-05

**Authors:** S. Koushesh, S.M. Shahtaheri, D.F. McWilliams, D.A. Walsh, M.N. Sheppard, J. Westaby, S.M. Haybatollahi, F.A. Howe, N. Sofat

**Affiliations:** † Institute for Infection and Immunity, St George's, University of London, Cranmer Terrace, London, SW17 ORE, UK; ‡ Pain Centre Versus Arthritis and NIHR Nottingham BRC, Academic Rheumatology, University of Nottingham Clinical Sciences Building, City Hospital, Hucknall Road, Nottingham, NG5 1PB, UK; § Molecular and Clinical Sciences Research Institute, St George's, University of London, Cranmer Terrace, London, SW17 ORE, UK; ‖ School of Psychology, University of Nottingham, University Park, Nottingham, NG7 2RD, UK

**Keywords:** Osteoarthritis, Bone marrow lesions, Magnetic resonance imaging, Histology, Grading, Scoring system

## Abstract

**Objectives:**

Bone marrow lesions (BMLs) are associated with pain in osteoarthritis (OA), but histological scores for OA focus on cartilage pathology. We developed a new scoring system, the Osteoarthritis Bone Score (OABS), to characterise OA-related BMLs.

**Methods:**

BML/non-BML tissues identified by Magnetic Resonance Imaging (MRI) in 10 knee OA subjects were harvested at total knee replacement (TKR). Osteochondral tissue from a further 140 TKR and 23 post-mortem (PM) cases was assessed. Histological features distinguishing MRI-defined BML/non-BML tissues on qualitative analysis were classified as present (0) or absent (1), summated for the OABS, validated by Rasch analysis and sensitivity to distinguish between sample groups. Immunohistochemistry for PGP9.5 assessed innervation.

**Results:**

Subchondral characteristics associated with BML tissues were cysts, fibrosis, hypervascularity, cartilage islands, trabecular thickening, loss of tidemark integrity and inflammatory cell infiltration. PGP9.5 immunoreactive perivascular nerves were associated with BMLs. OABS performed well as a measurement tool, displayed good reliability (Cronbach alpha = 0.68), had a 2-factor structure (trabecular/non-trabecular), with moderate correlation between the two factors (*r* = 0.56, 95% CI 0.46, 0.65). OABS scores were higher in TKR than PM cases with chondropathy, median difference 1.5 (95% CI -2, 0). OABS and Mankin scores similarly distinguished TKR from non-OA controls, but only OABS was higher in BML than non-BML tissues, median difference −4 (95% CI -5 to −2).

**Conclusions:**

OABS identifies and validly quantifies histopathological changes associated with OA BMLs. Histopathology underlying BMLs may represent 2 inter-related pathological processes affecting trabecular/non-trabecular structures. Increased vascularity/perivascular innervation in BMLs might contribute to pain.

## Introduction

Pain is one of the most prominent symptoms in osteoarthritis (OA). OA affects large numbers of people internationally, with a reported prevalence of over 30.8 million US adults alone suffering from OA^1^. It is now widely recognised that OA is a heterogeneous disease that affects several distinct components of the joint, including cartilage^2^, synovium^3^ and bone^4^. OA presents clinically across many endotypes e.g., post-traumatic, large joint, small joint or related to obesity and metabolic factors, each of which might involve different joint tissues to different extents. Methods that quantify changes observed in distinct joint compartments would help stratify OA by pathophysiology, and an improved understanding of OA pathophysiology will help lead to a deeper understanding of which mediators are involved in driving tissue damage and pain in the distinct joint compartments during OA.

Previous histological scoring systems have been developed primarily to quantify changes in articular cartilage in OA, such as the OARSI grade^5^ and chondropathy score reported by Mankin^6^. Other histological scoring methods for OA have focused on synovium, or, in subchondral bone on trabecular changes^10^. More recently, attempts have been made to establish the frequency of specific histological changes in cartilage, synovium and bone. Wyatt *et al.*^7^, using a repository of TKR and post-mortem (PM) tissues, identified discrete OA subgroups characterised by subchondral bone changes or synovitis.

Bone marrow lesions (BMLs) are a known feature of OA bone and their extent, as scored using Magnetic Resonance Imaging (MRI), is associated with OA pain^8^^,^^9^. However, histological changes in subchondral bone in OA BMLs are incompletely understood. BMLs are highly metabolically active structures, with features of angiogenesis, increased fibrous connective tissue, gene expression indicative of cartilage formation and bone resorption^11^^,^^12^. Muratovic *et al.*^13^ also showed increased bone matrix microdamage and altered vasculature in the bone of BMLs harvested at joint replacement surgery. BML innervation is currently unknown, but would be a prerequisite for BMLs to be a direct anatomical source of OA pain. Measurement of key histopathological features of BMLs would facilitate understanding of structure:symptom relationships, and help direct future therapeutic development aiming to both maintain joint structure and to improve pain in OA.

In the current study, we aimed to develop a novel and robust histological scoring system for human OA BML-like subchondral pathology and to explore BML innervation using PGP9.5-immunoreactivity^14^. Our findings extend understanding of underlying pathophysiology of BMLs, evaluated with the new Osteoarthritis Bone Score (OABS).

## Methods

In this study we analysed 10 cases for whom we had clinical and MRI data to define BML vs non-BML tissue by MRI co-localisation with tissue biopsies. We defined a semi-quantitative scale from seven typical histological features observed in these cases. We then tested this newly defined Osteoarthritis Bone Score (OABS) in a larger archive of OA tissue harvested at knee replacement surgery or post-mortem. Here we outline how the OABS performed at identifying bone/subchondral changes in OA vs control samples.

### Human osteochondral tissues

In order to characterise BML tissues, osteochondral samples were harvested with full informed consent as previously described^11^ from participants at TKR for knee OA within 6 weeks after undergoing MRI scan of the target knee (Ethical Approval was granted from the London Central REC, study number 12/LO/1970). The ≤6-week time period was chosen to minimise any changes in BMLs between imaging and tissue collection.

Regions within the tibia demonstrating BMLs were confirmed independently by two Consultant Radiologists (Dr Christine Heron and Dr Vivian Ejindu) using the MRI Knee Osteoarthritis Score (MOAKS)^8^; a semi-quantitative scoring system which shows particular refinement when scoring BMLs -providing regional delineation and grading across subchondral regions. Only the tibial non-cystic BML scores were considered when locating and dissecting lesions as the tibial plateau is the only structure which remains intact during surgery. During scoring the joint was divided into three equal medial and lateral sub-regions (anterior, central and posterior) and the sub-spinous sub-region identified by the tibial spines. The size of the lesion (0: none, 1: <33% of sub-regional volume, 2: 33–66% of sub-regional volume, 3: >66% of sub-regional volume) and number of BMLs per sub-region was used to identify the BMLs which had a non-cystic delineation. The percentage of volume of BML vs cyst (0: none, 1: <33%, 2: 33–66%, 3: >66%) was used to eliminate the cystic lesions from analysis. If a cyst was present without an associated BML the cyst was scored as a 0. Only lesions with a percentage volume score of three were considered for analysis. Each sub-region was graded for size of any cartilage loss (partial and full-thickness loss) (0: none, 1: <10% of region of cartilage and surface area, 2: 10–75% of region of cartilage surface area, 3: >75% of region of cartilage surface area). All images were given a score for the degree of synovitis (0 = normal; 1 = mild; 2 = moderate; 3 = severe).

**Fig. 1:**
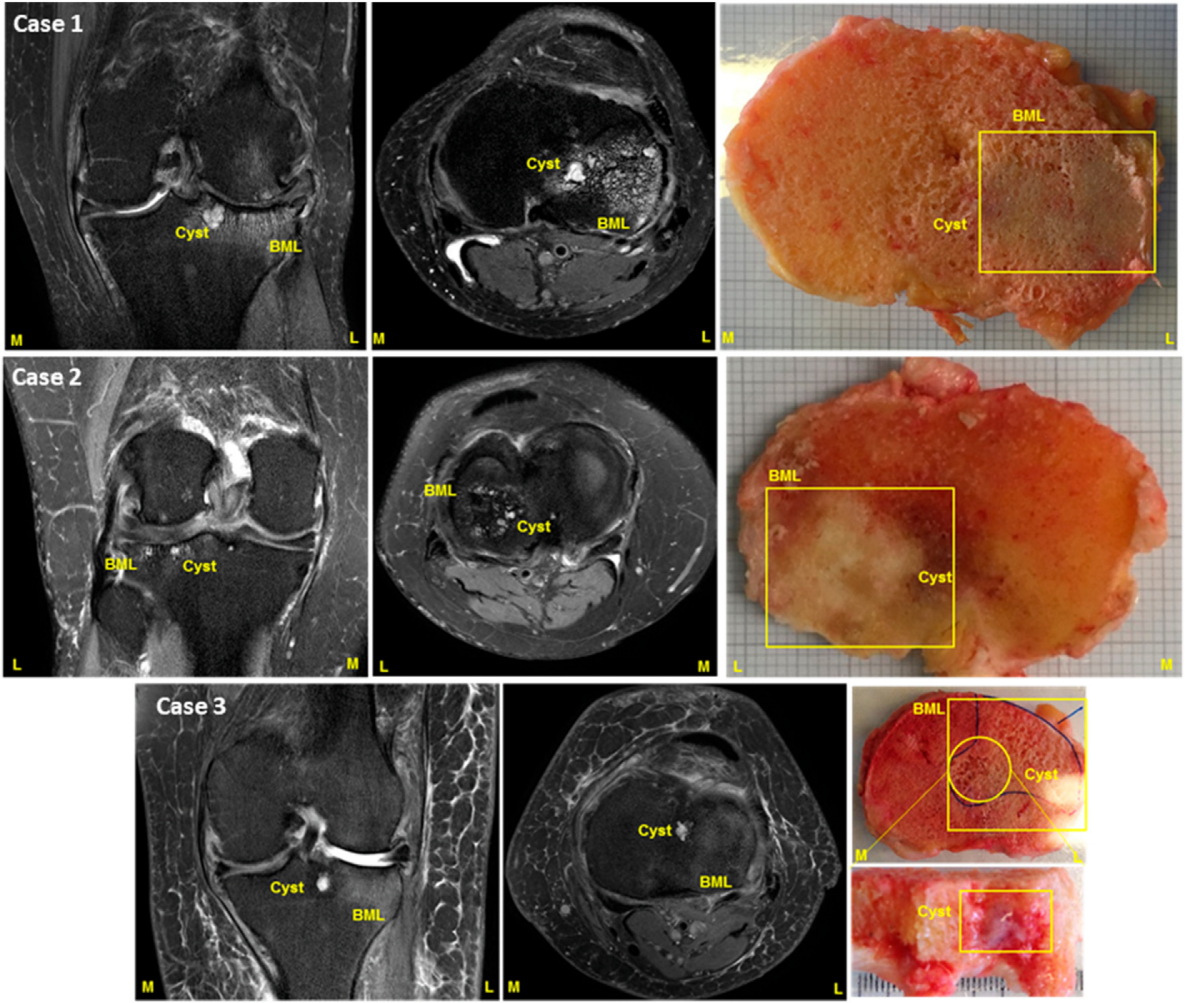
**Co-localisation of MRI Images and joint tissue obtained at surgery**. Representative images of Axial and Coronal views of target knee for co-localisation of knee biopsy tissue by MRI identification before tissue harvest at joint replacement surgery. MRI scans are shown alongside a macroscopic image of tibia for each subject harvested at the time of joint replacement. Each row represents an individual participant dataset for MRI and knee tissue. Yellow boxes represent BML tissue that was analysed. M: medial compartment; L: lateral compartment.

MRI data were acquired with a Philips 3T Achieva using a dedicated eight channel knee coil (Fig. 1). Pulse sequences were turbo spin echo acquisition of sagittal, coronal and axial intermediate-weighted (IW) images with TE 30 ms, TR 5000 ms, with Spectral Attenuated Inversion Recovery (SPAIR) fat-saturation and sagittal T1-weighted with TE 15 ms, TR 600 ms. Other parameters were: a) IW sagittal images with a 160 by 160 mm field of view (FOV) and 0.35 mm in plane resolution reconstructed to 0.25 mm resolution, acquired with thirty two 3 mm slices and 0.3 mm slice gap; the turbo spin echo sequence had 30 echoes acquired with SENSE factor 1.6 leading to a 4′ 50″ acquisition time; b) IW coronal images with a 160 by 160 mm FOV and 0.35 mm in plane resolution reconstructed to 0.25 mm resolution acquired with twenty-two 3 mm slices and 0.3 mm slice gap; the turbo spin echo sequence had 30 echoes acquired with SENSE factor 1.4 leading to a 4′ 50″ acquisition time; c) IW coronal images with a 160 by 160 mm FOV and 0.35 mm in plane resolution, reconstructed to 0.2 mm resolution, acquired with thirty-two 3 mm slices and 0.25 mm slice gap; the turbo spin echo sequence had 20 echoes acquired with SENSE factor 1.4 leading to a 5′ 30″ acquisition time; c) T1w sagittal images with a 150 by 137 mm FOV, 0.3 mm in plane resolution reconstructed to 0.15 mm resolution, acquired with thirty-five 3 mm slices and 0.25 mm slice gap; the turbo spin echo sequence had eight echoes acquired with SENSE factor 1.6 leading to a 6′ 34″ acquisition time. Within the IW images, SPAIR fat-saturation was applied with the manufacturer's parameters of “strong” fat-suppression with a power level one and an automatically determined inversion time.

### Co-localisation with MRI scans and tissue biopsy

The Orthopaedic Surgeon provided a bespoke procedure during joint replacement surgery by harvesting the intact tibial plateau. After removal, BML and non-BML tissue were approximately co-localised by the surgeon with MOAKS-defined BMLs, labelled with a marker for further dissection and processing. In addition to BML regions, a comparator non-BML region from the same participant was also labelled. Fig. 1 illustrates co-localisation of MRI images and pathological samples. The 10 BML and 10 non-BML samples were used to develop the OABS histological scoring system. For sample processing please see Supplementary methods.

In order to then further develop and validate the histological scoring system in an independent dataset of subchondral bone samples, tissue was assessed in post-mortem samples and subchondral bone from knee joint replacement surgeries. Subchondral bone from the mid-coronal section of medial tibial plateau from additional cases were donated without MRI, at TKR for OA, or post mortem, as previously described (Ethical Approvals from the East Midlands Derby REC, study number 16/EM/0026 and Nottingham Research Ethics Committee 1 (05/Q2403/24) and Derby Research Ethics Committee 1 (11/H0405/2)^7^. In the development study, non-arthritic post mortem cases had no known history nor macroscopic appearances of OA, were age and sex matched to OA cases, and had not undergone knee MRI. In the validation study, TKR and post mortem cases were consecutive cases from the Sherwood Forest Hospitals NHS Foundation Trust Joint Tissue Repository.

### Tissue preparation histological analysis

Tissue sections were stained with haematoxylin and eosin or Safranin O/Fast green using standard protocols (one of each per case). A standardised chondropathy score for each participant was obtained using the Mankin chondropathy grading system (0–14)^6^.

Subchondral features associated with BMLs were identified using an iterative process of analysing the histological changes observed independently (by SK, NS and MS).

There were five groups of histological samples tested in our study, namely Group 1: TKR for OA (development set with MRI co-localisation for BML and histology); Group 2: Post-mortem non-arthritic controls (development set); Group 3: TKR for OA (development set); Group 4: TKR for OA (validation set); Group 5: Post-mortem samples with chondropathy (validation set).

**Fig. 2:**
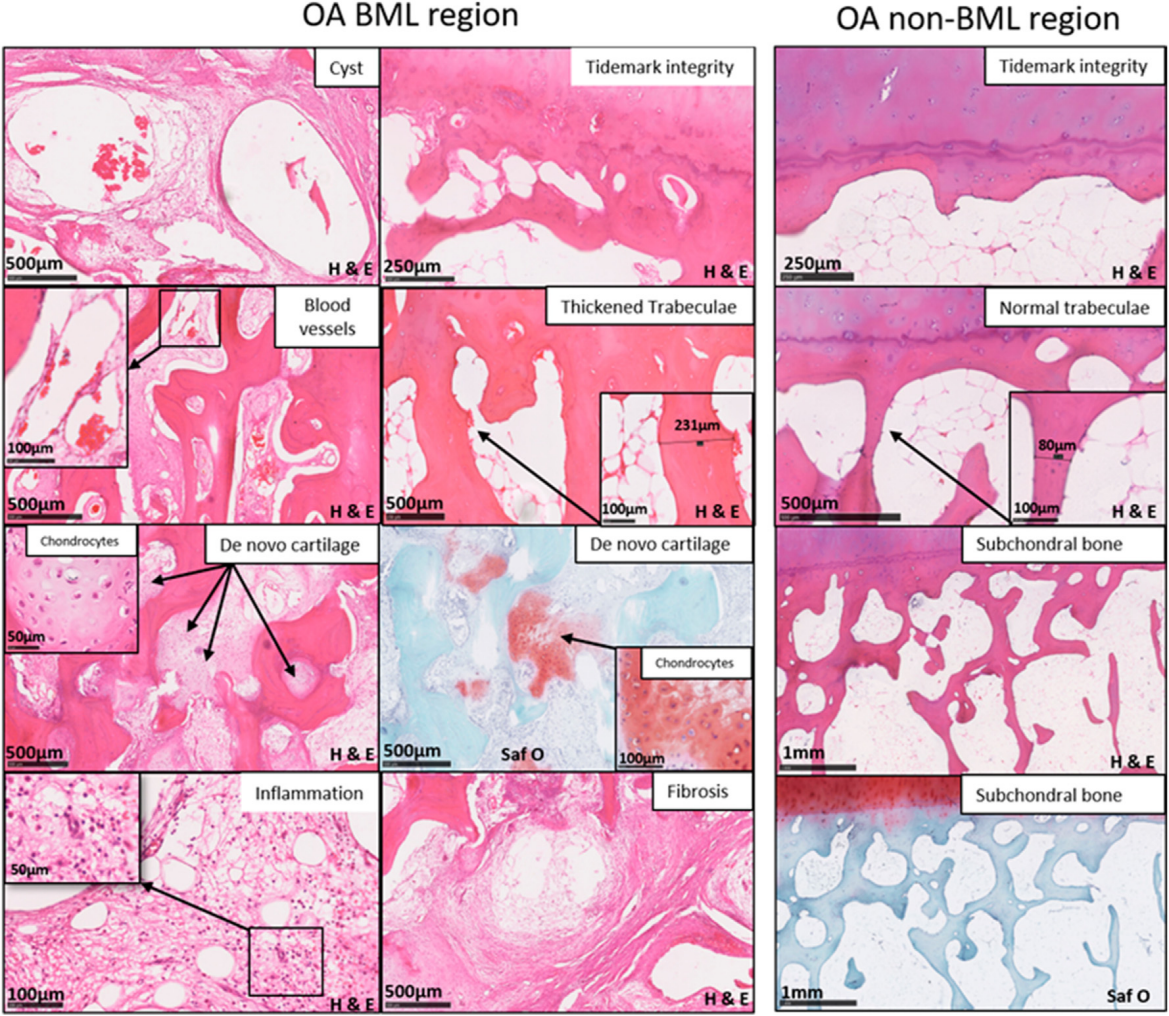
**Histological features of bone marrow lesions comprising the Osteoarthritis Bone Score (OABS)**. Appearances of the seven histological characteristics of BML regions, each scored as present (1) or absent (0) to constitute the OABS. Characteristic tissue changes are present within BML samples, but absent from the illustrated non-BML OA samples. Haematoxylin (blue) eosin (pink) or Safranin O (pink) and Fast Green staining. Typical histological changes shown in OA BML panel indicated by arrows are cysts, fibrosis, hypervascularity, cartilage islands (appears pink on Safranin O stain), thickened trabeculae, loss of tidemark integrity at the cartilage/bone interface and inflammation with increased presence of macrophages and osteoclasts. The panels on the right show non-BML OA tissue. Whole slide scanning with Nanozoomer technology is shown.

**Fig. 3:**
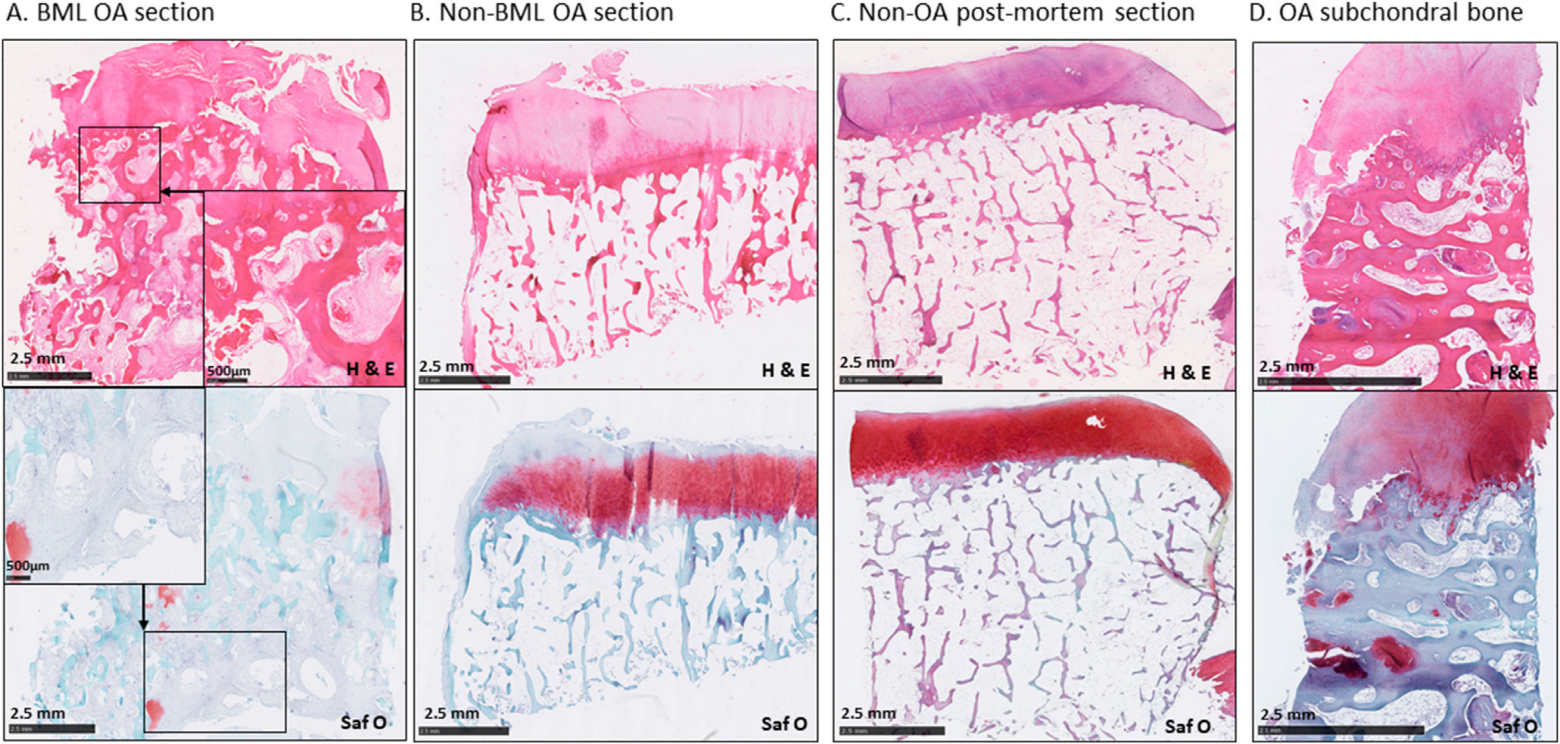
**Osteochondral histopathology in osteoarthritis**. Representative stained sections of articular cartilage and subchondral bone showing BML (A) and non-BML (B) regions, localised by reference to preoperative MRI scans, and a sample from a patient without prior imaging (D), each from patients who underwent total knee replacement surgery for OA. Non-OA post-mortem control tissue is shown in (C). Subchondral pathology is most evident in regions identified as BMLs, and associated with more severe pathology in the overlying articular cartilage. Upper panels are stained with haematoxylin and eosin, with lower panels stained with Safranin O/Fast Green.

Seven domains of pathological change were identified associated with BMLs; cysts, subchondral fibrosis, hypervascularity, cartilage islands indicating new cartilage within the bone, thickened trabeculae, breaching of the tidemark between the cartilage and bone interface by blood vessels, and cellular infiltrates suggesting inflammation (Fig. 2, Fig. 3).

Each feature was categorised as 0 (absent) or 1 (present) in order to develop a simple, semiquantitative scoring method, suitable for application using readily available laboratory equipment (Table I). Except where otherwise stated, each feature was assessed within a standardised depth of tissue 7.2 mm from the osteochondral junction into the bone and width of 15.5 mm was analysed for each section. Trabecular thickness was evaluated by reference to microscopic field size in any trabeculae within 1 mm below the chondro-osseous junction^15^. Trabecular thickening was classified if trabecular thickness was greater than 200 μm at its widest point in at least two trabeculae per section per case. Inflammation was classified as hypercellular fibrovascular infiltration of marrow spaces in at least one region per section, containing any or all of the following: macrophages, lymphocytes, neutrophils, eosinophils, plasma cells, or histiocytes, with or without multinuclear osteoclasts. Osteoclasts were identified in haematoxylin and eosin stained sections by the presence of multinucleated giant cells. It is appreciated that the accuracy of identifying osteoclasts by H&E and not TRAP staining has some limitations. Each BML-associated feature was scored 0 (absent) or 1 (present) and a total Osteoarthritis Bone Score (OABS) was derived by summing individual characteristics. The OABS system was applied to all sections (Table I, Table II, Fig. 2).

**Table I:** Summary of Osteoarthritis Bone Score with seven domains and grading

Osteoarthritis Bone Score (OABS)	Grade
1. Cysts	
None	0
Present (at least 1)	1
2. Fibrosis (fibrotic connective tissue within bone marrow space)	
None	0
Present (at least one region)	1
3. Blood vessels (number of blood vessels within the subchondral region of interest)	
Normal (0–15)	0
Increased (>16)	1
4. Cartilage islands (new cartilage within bone)	
Absent	0
Present	1
5. Trabeculae thickened (≥2 trabeculae >200 μm wide)	
Normal	0
Increased thickness	1
6. Tidemark Integrity	
Intact	0
Crossed by at least one blood vessel	1
7. Inflammation (cellular infiltrates)	
Absent	0
Present	1
Total	7

Except where otherwise stated, each feature was assessed within a standardised depth of tissue 7.2 mm from the osteochondral junction into the bone and width of 15.5 mm in each section. The grade of 0/1 was used for each category to have a simple and semiquantitative scoring/classification method.

**Cyst:** a thin-walled cavity containing fluid.

**Fibrosis**: Presence of fibrous connective tissue within bone marrow space.

**Blood vessels**: Annular or linear structures containing smooth muscle or red cells.

**Cartilage islands**: Extracellular matrix staining pink by Safranin O or eosin, containing chondrocyte-like mononuclear cells.

**Trabecular thickening**: at least two trabeculae per section with trabecular thickening defined as > 200 μm at its widest point. Trabecular thickness was evaluated within 1 mm below the chondro-osseous junction.

**Tidemark integrity**: presence of at least once blood vessel breaching the tidemark.

**Inflammation**: hypercellular fibrovascular infiltration of marrow spaces in at least one region per section, containing any or all of the following: macrophages, lymphocytes, neutrophils, eosinophils, plasma cells or histiocytes.

**Table II:** Demographics, clinical and histopathology scores in developmental study groups

Outcome measure	Developmental set (groups 1–3)				Validation set (groups 4, 5)	
	TKR for OA (Group 1)		Non arthritic controls (Group 2)	TKR for OA (Group 3)	TKR for OA (Group 4)	PM with chondropathy (Group 5)
	BML	non-BML				
Number	10		10	20	140	23
Female; N (%)	9 (90)		7 (70)	13 (65)	77 (55)	10 (43)
Age; mean (SD)	67.8 (9.2)		64.5 (5.5)	67.9 (7.8)	65.5 (9.7)	67.1 (14.9)
Mankin chondropathy score						
(mean, SD)	9.9 (3.4)	7.2 (4.5)	2.7 (1.7)	8.3 (4.0)	10.5	9.4
Median (IQR)	11 (8–12)	6 (4–13)	3 (1–4)	8 (5–13)	11 (9–11)	9 (7.5–11)
OABS						
(mean, SD)	6.2 (1.5)	2.9 (1.2)	1.1 (0.6)	5.4 (2.1)	5.3 (1.3)	4.3 (1.5)
Median (IQR)	7 (6–7)	3 (2–4)	1 (1–1)	7 (3–7)	6 (4–6)	4 (3–5)
Blood vessel density Per section (mean, SD)	123.5 (69.1)	53.2 (21.4)	11.7 (5.4)	n/a	n/a	n/a
Blood vessel (+) PGP9.5 (mean, SD)	15.5 (13.2)	4.4 (5.3)	0.4 (0.69)	n/a	n/a	n/a

Study data summarising age, sex, Osteoarthritis Bone Score (OABS) and Mankin chondropathy score for sample population. There were five groups in the study: Group 1: TKR for OA (main development set with MRI co-localisation for BML and histology), Group 2: Post-mortem non-arthritic controls, Group 3: TKR for OA (development set), Group 4: TKR for OA (validation set); Group 5: Post-mortem samples with chondropathy (validation set).

Pain scores measured in Group one were WOMAC (Western Ontario and McMaster Universities Osteoarthritis Index), and VAS (Visual Analogue Scale) pain. Values are presented as mean and standard deviation (SD), or median and 25^th^ and 75^th^ percentiles (IQR).

For Group 1, mean (SD) BMI 30.9 kg/m^2^ (3.2), WOMAC pain score mean (SD) 57.7 (14.4), range 31.6–78.2, and pain VAS 5.8 (1.7) range 2.6–7.6, indicating moderate to severe pain.

Within the validation data set, OABS and Mankin scores each was similarly associated with TKR (group 4), post mortem with chondropathy (group 5) OABS; Odds ratio (OR) = 2.22 (95% CI, 1.41 to 3.64), *r* = 0.29, Mankin score; OR = 1.64 (95% CI, 1.06 to 2.58) giving Spearman's *r* = 0.29 for OABS and *r* = 0.19 for Mankin scores, difference between coefficients = 0.10 (95% CI -0.09 to 0.30, *p* = 0.284).

Blood vessel density shows the number of blood vessels counted per standardised subchondral region of interest for each subject (depth 7.2 mm and width 15.5 mm).

Abbreviations used: BML: Bone Marrow Lesion; non-BML: non-Bone Marrow Lesions, IQR; interquartile range, PGP9.5: Protein Gene Product 9.5; TKR: Total Knee Replacement; PM: post-mortem.

The OABS was developed using samples from 40 subjects (Groups 1–3) and validated with samples from 163 subjects (Groups 4–5). OABS development used paired samples of BML and non-BML tissue obtained at TKR from 10 people with OA (Group 1), and from 10 non-lesional samples from post-mortem control subjects (Group 2) with no known clinical history nor macroscopic bone or cartilage appearances of OA^2^. Samples from a further 20 participants without MRI were collected at TKR for OA to assess if histological changes defined from Group one were detectable in TKR samples from people with knee OA who had not been selected according to MRI appearances (Group 3) (Fig. 3). All developmental cases were scored by at least two readers (SK and NS) and blinded to diagnostic group. After scoring, a final consensus score was reached following review of histology items for which there was any disagreement by the two readers. For validation of OABS, tissue samples were scored (by MS) from a further 163 cases (140 TKR for OA (group 4) plus 23 post-mortem samples with macroscopic OA appearances; osteophytes (22/23), grade III chondropathy (19/23) and/or grade IV chondropathy (9/23) (Group 5)^16^. Psychometric properties were determined by Rasch analysis, and ability to discriminate between TKR and PM cases was determined.

### Subchondral vascular density and innervation

Number of blood vessels within subchondral bone spaces were counted manually to a depth of 7.2 mm from the articular surface across 15.5 mm of one haematoxylin and eosin-stained section per sample. Number of blood vessel profiles associated or not associated with PGP9.5 immunoreactive nerves in subchondral bone were counted manually in sections stained for PGP9.5 using the DAB substrate kit (ab64238) without enhancer and counterstained with haematoxylin (see supplementary methods).

### Statistical analysis

We used GraphPad Prism 11 (San Diego, California, USA) for descriptive, comparative and regression analyses. Demographic data were presented as mean and standard deviations, median and interquartile range. A priori planned comparisons were made between TKR BML and non-BML regions using Wilcoxon signed-rank test, and between TKR OA cases and non-OA controls (groups 2, 3) and between TKR and PM cases with chondropathy (groups 4, 5) using Mann Whitney U. Vascular densities were compared across groups 1–3 using Kruskall Wallis test. Associations of case group (groups four *v* five) with OABS or Mankin chondropathy score are expressed as Odds Ratios (OR) from logistic regression, and strengths of association compared using Spearman's rank correlation coefficients. Comparisons between coefficients used Pearson correlations within the cocor package in *R* software, the analysis was adjusted for the overlapping dependency of the coefficients with r-to z-transformations to address non-normality of data.

Factor analysis for binary data was undertaken to test OABS structure following creation of a polychoric correlation matrix, using Stata^17^. Promax rotation allowed correlation of emergent factors from dichotomous items within OABS. Principal component analysis (PCA) examined the contribution of each domain to overall subchondral pathology and the factor structure of OABS. Rasch analysis examined measurement properties of the OABS score using RUMM2030 software (http://www.rummlab.com.au/). Model fit was assessed by comparing the means and SD of items' and persons' (cases’) locations with the theoretical values (see supplementary data). Internal consistency was measured using Cronbach alpha.

## Results

### Histopathological characteristics of bone marrow lesions

**Fig. 4:**
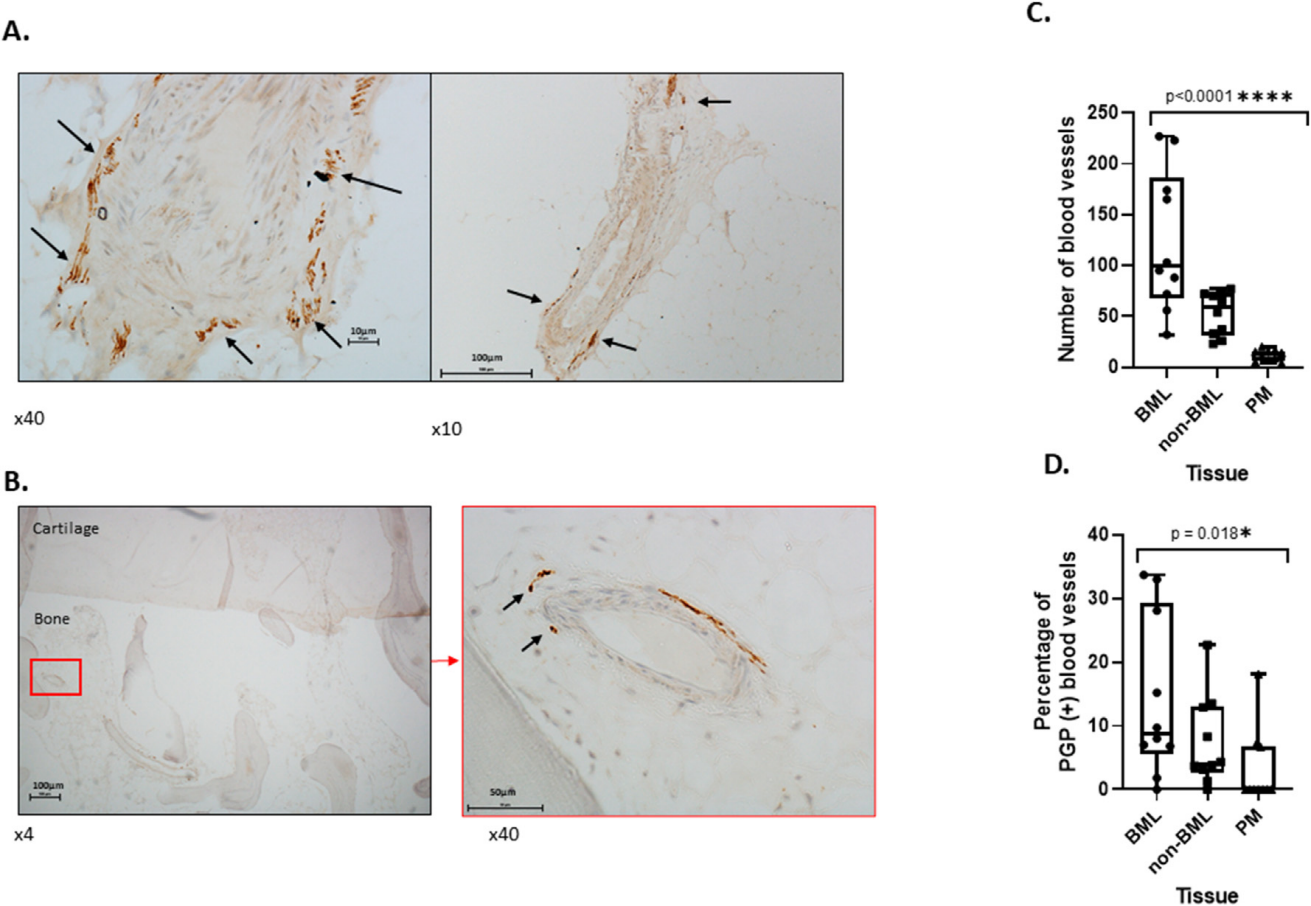
**Innervation of Bone Marrow Lesions. A:** Nerve profiles in fibrotic OA BML tissue and perivascular staining. **B:** Perivascular PGP 9.5 immunoreactive nerves within subchondral BML bone. **C:** Box plots of subchondral vascular density in BML and non-BML OA samples, and in non-arthritic post-mortem (PM) controls. Number of blood vessels differed significantly between the three groups (Kruskall Wallis test, *p* < 0.0001). **D:** Box plots of percentage of blood vessels associated with PGP 9.5-immunoreactive nerves in subchondral bone of BML and non-BML OA samples (group 1), and in non-arthritic PM controls (Group 2). Percentage of blood vessels associated with PGP9.5 immunoreactivity differed significantly between the three groups (Kruskall Wallis test, *p* = 0.018). Box plots show maximum, minimum and IQR range of values. Nerves were visualised by immunoreactivity for PGP9.5 and counterstained with haematoxylin. To obtain the total number of blood vessels in the standardised subchondral region of interest (see methods), each blood vessel was counted by whole slide scanning using Nanozoomer technology.

Table II gives participant details. Individual participant data for MRI MOAKs scores and bone pathology characteristics for Groups one and two are in Supplementary Table 1. Hypervascularity in BML tissue was most frequently observed in regions near the osteochondral junction, with other regions of increased blood vessels deep within the subchondral bone. Subchondral vascularity was higher in BML tissue 123.5 (SD 69.1) compared with non-BML tissue 53.2 (SD 21.4) and post-mortem controls 11.7 (SD 5.4) *p* < 0.0001 [Fig. 4(C)]. PGP9.5 immunoreactive nerve profiles were most frequently observed in a perivascular distribution at the osteochondral junction and deeper within subchondral bone [Fig. 4(A) and (B)]. Mean percentage of blood vessels associated with PGP9.5 immunoreactivity was higher in BML (15.5, SD 13.2%) than in non-BML OA (4.4, SD 5.3%) or non-OA control (0.4, SD 0.69%) tissues, *p* = 0.018 [Fig. 4(D)].

### Development and internal validation of Osteoarthritis Bone Score

Each of the 7 BML-associated features was classified as absent (0) or present^1^, and summed to give an OABS ranging from 0 to 7, with 0 showing no pathological change and seven the greatest pathological change (Fig. 2). We used Rasch analysis of OABS across all 203 cases, to investigate if our scoring system complies with the principles of measurement development (Supplementary Tables 1 and 2). Item location fit was good (mean 0, SD 1.251), with the Cysts item displaying highest difficulty (location = 2.211) and Trabeculae thickened the lowest difficulty (location = −1.575). Mean person (case) location was 1.196 (SD 1.430), close to the expected theoretical value of one for a good person location fit. The mean person fit residual (−0.225) was closer to the ideal value of 0 than was the item fit residual (−0.445), although with a high degree of uncertainty. Floor and ceiling effects for OABS were acceptable. Only one person (0.49%) had a score of 0 in all items, although 45 samples (22%) had an OABS score of 7.

Principal Component Analysis (PCA) examined OABS structure and the contribution of each domain to subchondral pathology. Polychoric correlation matrix indicated positive correlations between almost all item pairs (Supplementary Table 3). The seven OABS items were significantly loaded on two factors (Eigenvalues 3.1 and 1.2) (supplementary Tables 2 and 4). All items except trabecular thickness were loaded on the first factor (loadings 0.47 to 0.91) (Supplementary table 5), and the second factor included only one item: trabecular thickness (supplementary Tables 3 and 6). The first factor explained 66%, and the two factors cumulatively explained 92% of the OABS variance. The two factors were intercorrelated (*r* = 0.56), and Cronbach alpha for OABS was 0.676.

### Discriminative ability of OABS and mankin chondropathy scores

**Fig. 5:**
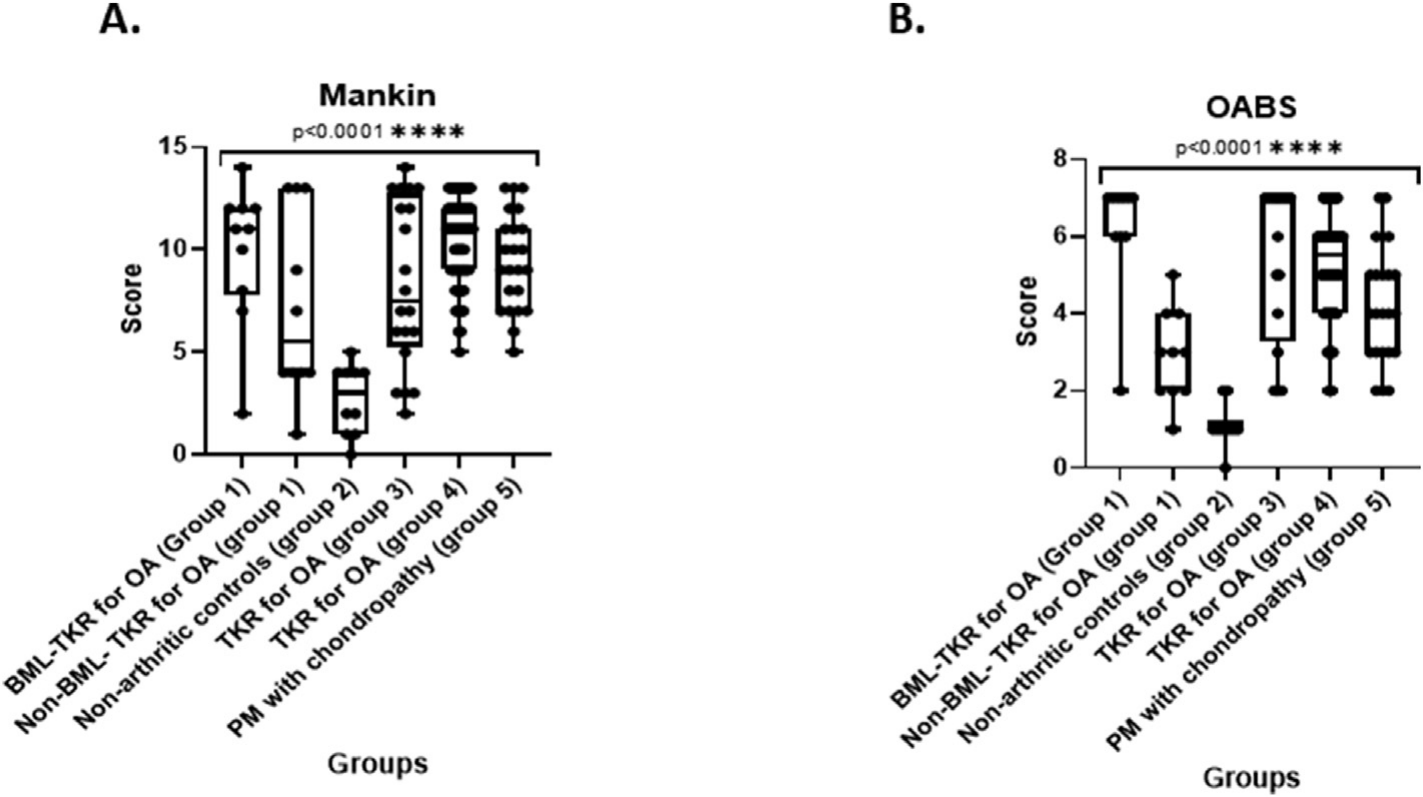
**Box plots of Osteoarthritis Bone Scores and Mankin chondropathy scores**. **A:** Box plots showing distribution of OABS scores in case groups corresponding to Table II. **B:** Box plots showing distribution of Mankin scores in case groups corresponding to Table II. Horizontal bars indicate median, IQR, maximum and minimum range of values.

Mean OABS in BML regions (6.2, SD 1.5) was higher than in non-BML regions (2.9, SD 1.2) from OA cases (*p* = 0.0039) and OABS in TKR cases (group 3; 5.4, SD 2.1) was higher than in age and sex matched non-arthritic controls (group 2; 1.1, SD 0.6, *p* < 0.0001) (Table II, Fig. 5). OABS in TKR OA cases were similar to BML lesion samples. OABS discriminated group 4 (OA TKR cases) from group 5 (post mortem chondropathy cases who had not undergone TKR) (Table II).

OABS was significantly associated with Mankin chondropathy scores across all cases (Spearman's *R* = 0.79, *p* < 0.0001), although high Mankin scores were observed both in the BML and in non-BML OA samples (Table II, Fig. 5). As with OABS, Mankin scores discriminated BML TKR OA cases (Group 1) from non-arthritic post-mortem controls (Group 2) (*p* = 0.0003), and TKR from post mortem chondropathy cases (*p* = 0.038) (Table II). However, unlike with OABS, there was no significant difference in Mankin scores between BML and non-BML samples (*p* = 0.22).

## Discussion

In this study we identify seven histopathological characteristics of BMLs; cysts, fibrosis, hypervascularity, cartilage islands, trabecular thickening, loss of tidemark integrity and inflammatory cell infiltration, and validate a composite scoring system, the OABS. BML-like histopathological changes were associated with subchondral hypervascularity and innervation, consistent with a highly metabolic tissue change that is associated with OA pain.

Robust scoring systems for pathology in distinct compartments of the joint can help elucidate disease mechanisms and support disease classification. Although validated scoring systems exist for synovial changes^3^ and cartilage damage^5^^,^^6^, previous histological measurement of bone pathology has focused on calcified structural components (e.g., trabecular morphology and subchondral bone thickness)^10^, whereas cellular and metabolic changes may make greater contributions to symptoms such as pain. In this respect, histological correlates of BMLs are of particular interest because MRI evidence of BMLs has been consistently associated with OA pain^8^^,^^9^. We have shown that the subchondral pathological features can be scored using the OABS. OABS was well targeted to a broad population of donors of tibial plateaux at TKR or post mortem, of whom only 22% displayed changes given the maximum score of 7 (ceiling effect). OABS could distinguish BML lesions from non-BML subchondral bone, and could distinguish between OA and non-OA cases, and between samples obtained at TKR or post mortem. The OABS may be used in conjunction with established scoring systems for synovium and cartilage^3^^,^^5^^,^^6^ to quantify structural changes in the three discrete tissue compartments of the OA joint, each of which might be a source of OA pain.

As with MRI-based studies in knee OA, we found that areas of more extensive histological cartilage damage were associated with areas of BML-like subchondral pathology^18^. Zanetti *et al.* determined histologically that BMLs may contain normal fatty marrow with marrow necrosis, necrotic or remodelled trabeculae, oedema and extravasation of blood within bone marrow spaces^19^. A longitudinal MRI study in knee OA showed that higher baseline BMLs size was strongly associated with progressive loss of cartilage^20^. Reciprocally, Roemer *et al.* found that baseline cartilage loss was associated with subsequent BML^21^.

The mechanisms by which BMLs develop are incompletely understood, and their focal association with cartilage pathology might indicate induction by pathological cartilage products such as matrix fragments^22^, from abnormal stresses through subchondral bone under defective cartilage, from the infiltration of synovial fluid through chondral defects contributing to form subchondral cysts, or to impairment of cartilage homeostasis due to disrupted structural or metabolic support from subchondral bone^22^.

Subchondral and cartilage changes are both components of OA pathology. Accordingly, OABS scores were associated with high Mankin chondropathy scores, and both OABS and Mankin scores could distinguish between OA cases and non-OA controls, and between surgical and non-surgical chondropathy cases. However, OABS scores differed between BML and non-BML regions from OA tibial plateaux, to a greater extent than did Mankin scores for the overlying cartilage. This suggests that OABS reflects a specific feature of OA pathology which is not simply a consequence of chondropathy.

Our findings contribute to the understanding of why BMLs are associated with pain in OA by demonstrating hypervascularity and innervation in BMLs. We extend previous findings of sensory nerves within bone and at the osteochondral junction, which may be associated with OA pain^23^^,^^24^, to show that BMLs are innervated. Associations between MRI-detected BMLs and pain may therefore be directly mediated by nerves within BMLs. Hypervascularity might represent angiogenesis, as also suggested by angiogenic pathway gene expression within BMLs^10^. Microarray studies in OA BML tissue have also shown upregulation of genes implicated in neurogenesis^11^. Nerve growth often accompanies vascularisation, such that hypervascularity and neoinnervation may be co-regulated in BMLs^24^. BMLs also contain inflammatory cells, including macrophages and osteoclasts, suggesting that the demonstration by Aso *et al.*^12^ of macrophage infiltration and osteoclast in nerve-rich tissue within OA subchondral bone may reflect the same subchondral pathology now identified as BMLs. Based on our findings, we propose that increased inflammatory cell infiltration, coupled with angiogenesis and neoinnervation at sites of increased osteoclast activity, together are mediators of BML-induced OA pain.

Our study was subject to several limitations. We investigated only human knee samples, and it is possible that BML tissue has other distinct pathological changes in other regions e.g., hip, foot or hand, anatomical regions that are subject to different loading, biomechanical and structural factors. We developed OABS using samples from people with severe OA requiring knee replacement surgery, and, even though we included age- and sex-matched controls and validated OABS in a broad range of TKR and post mortem cases, it is possible that additional pathological features may be present in early BMLs. Our data indicate a good fit to the Rasch model necessary for a valid measurement tool, and OABS could distinguish between the majority (78%) of samples. However, nearly one quarter of the samples had maximum OABS scores of 7. Future research might reveal additional aspects of BML pathology which complement the seven characteristics included within OABS and better distinguish between extreme cases. Future research may explore the temporal progression of BML formation during different stages of OA. We used immunohistochemistry for PGP 9.5, which may lack sensitivity and specificity for subchondral sensory nerves, and our data should be considered an index of subchondral innervation, rather than an absolute measurement. Other markers specific for sensory rather than sympathetic nerves might extend our current findings, although limits of sensitivity of immunohistochemistry in decalcified human tissues may lead us to underestimate the true density of innervation. We acknowledge that the tibial samples in our study are only a portion of BML tissue, which could be extending deeper into subchondral bone, therefore only part of BML tissue was evaluated. Individual histological changes such as cartilage islands or trabecular thickness, may each contribute to similar MRI appearances but respond differently to treatment, and OABS may not provide a complete picture of changes in pathophysiology.

There is currently a desperate need to find treatments that can modulate structural damage and pain in OA. Pathological changes in OA cartilage and synovium have been well-described^3^^,^^5^^,^^6^. In recent years, several studies have tested effects of new potential therapeutic agents on cartilage/synovium structure and function. Examples include biologic agents such as canakinumab^25^, non-steroidal anti-inflammatory agents^26^, fibroblast growth factor-18 receptor 3 (Sprifermin)^27^ and wnt pathway modulators such as lorecivivint^28^. However, effects on human bone pathology and OA symptoms have not to date been clearly demonstrated. Our new OABS scoring system could be developed as a biomarker to better understand possible mechanisms through which BMLs occur, and why they might cause pain or structural damage. OABS might be amenable to back translation into animal models to further elucidate subchondral OA mechanisms and develop novel therapeutics. OABS and further exploration of subchondral pathology should complement progress in understanding cartilage and synovial biology to accelerate the development of more effective treatments that will relieve the great burden of OA on individuals and society.

## Contributions

SK and SSM prepared and analysed tissue samples for histology and immunohistochemistry and conducted data analysis. NS wrote the initial study protocol and associated documents, co-ordinated the implementation of the study, assisted with data analysis and drafted the manuscript. MNS and JW contributed to processing and histology with help in analysis of the histological and innervation changes. SMS, DM, DAW, MNS, JW, FAH and SH contributed to the final study design, data acquisition/analysis, writing and review of the manuscript.

## Conflict of interest

There are no conflicts of interest related to this study.

## Funding

Supported by the Rosetrees’ Trust (Grant number M11-F3), the Wellcome Trust Institutional Support Fund (ISSF) (Grant number 204809/16/Z), the National Institute for Health Research (NIHR) Clinical Research Network, the NIHR Nottingham Biomedical Research Centre, and Versus Arthritis (Grant number 20777). The views expressed are those of the author(s) and not necessarily those of the funders.
