# Supplementary material for: The osteoarthritis bone score (OABS): a new histological scoring system for the characterisation of bone marrow lesions in osteoarthritis
Source: Osteoarthritis Cartilage. 2022 May;30(5):746–55. doi: 10.1016/j.joca.2022.01.008 (PMC9395274; doi:10.1016/j.joca.2022.01.008)
Supplement: Multimedia component 1 [file mmc1.docx]

**Supplementary methods**

*Methodology for Co-registration of MRI scans with tissue for biopsy*

MRI scans of all target knees were taken pre-operatively (up to 3-6 weeks’ pre-operatively) and used to localise BMLs within the subchondral bone. The 3-6-week time period was decided upon as BMLs are subject to change in size and location after 6 weeks (Felson et al, 2012). Axial (Ax), coronal (Cor) and sagittal (Sag) images were acquired with a Philips 3T Achieva system using a dedicated 8 channel peripheral knee coil for small/medium knees and a flex wiresystem for larger knees (Figure 1). Pulse sequences were turbo spin echo acquisition of sagittal, coronal and axial intermediate-weighted (IW) images with echo time (TE)30ms, repetition time (TR).

MRI knee scans were performed with Spectral Attenuated Inversion Recovery (SPAIR) fat-saturation and sagittal T1-weighted with TE 15ms, TR 600ms.Peripheral knee coil sequence: repetition time TR) 5000ms, echo time (TE) 30ms, Field of view (FOV) 160mm, Ax & Cor matrix 0.35 x 0.35, slice thickness 3mm with a 0.3mm interslice gap; Sag matrix 0.35 x 0.35, slice thickness 2.5mm with a 0.25mm interslice gap. Flex wire system sequence: repetition time (TR) 5000ms, echo time (TE) 30ms, Field of view (FOV) 160mm, Axial & Coronal matrix 0.5 x 0.5, slice thickness 4mm, interslice gap 4mm; Sag matrix 0.5 x 0.5, slice thickness 3.5mm, interslice gap 3.5mm).

Other parameters were:

a) IW sagittal images with a 160 by 160 mm field of view (FOV) and 0.35mm in plane resolution reconstructed to 0.25mm resolution, acquired with thirty-two 3mm slices and 0.3mm slice gap; the turbo spin echo sequence had 30 echoes acquired with SENSE factor 1.6 leading to a 4’ 50” acquisition time.

b) IW coronal images with a 160 by 160 mm field of view (FOV) and 0.35mm in plane resolution reconstructed to 0.25mm resolution acquired with twenty-two 3mm slices and 0.3mm slice gap; the turbo spin echo sequence had 30 echoes acquired with SENSE factor 1.4 leading to a 4’ 50” acquisition time.

c) IW coronal images with a 160 by 160 mm field of view (FOV) and 0.35mm in plane resolution, reconstructed to 0.2mm resolution, acquired with thirty-two 3mm slices and 0.25mm slice gap; the turbo spin echo sequence had 20 echoes acquired with SENSE factor 1.4 leading to a 5’ 30” acquisition time.

d) T1-w sagittal images with a 150 by 137 mm field of view (FOV), 0.3mm in plane resolution reconstructed to 0.15mm resolution, acquired with thirty-five 3mm slices and 0.25mm slice gap; the turbo spin echo sequence had 8 echoes acquired with SENSE factor 1.6 leading to a 6’ 34” acquisition time. Within the IW images, SPAIR fat-saturation was applied with the manufacturer’s parameters of “strong” fat-suppression with a power level 1 and an automatically determined inversion time.

*Preparation of BML tissue*

Ten participant samples (tibial plateau) were collected and specimens were fixed in 10% (v/v) neutral buffered formalin for 24 hours then decalcified using formic acid containing 40% (v/v) formalin. To overcome difficulties in studying the morphology of joint tissue, specimens were placed in the decalcification solution at 20 times the tissue volume for up to 14 days at room temperature. Samples were dehydrated in graded alcohol series and paraffin embedded before being sectioned using a microtome at 5µm (Leica RM2255, Milton Keynes, UK). To assess morphology, formalin-fixed sections were stained with haematoxylin and eosin (H&E) and Safranin-0 Fast Green to visualise proteoglycan content and presence of *de novo* cartilage. Slides were scanned using a NanoZoomer 2.0-RS Digital Scanner at 20X (Hamamatsu, Hertfordshire, UK) and visualised with the NanoZoomer Digital Pathology v2.0 software (Hamamatsu, Hertfordshire, UK).

*Tissue preparation for histological scoring*

Tissue sections were stained with haematoxylin and eosin (manufacturer) and Safranin O/Fast green (manufacturer) protocols. A standardised chondropathy score for each participant was obtained using the Mankin chondropathy grading system (0-14) (6). Then, scoring by the newly developed Osteoarthritis Bone Score (OABS) system was applied to all sections. Scores of 0 (normal) or 1, indicating presence of changes corresponding to the domain involved, were used to maintain simplicity and clarity of the scoring system. We included 7 domains comprising the structure of bone (cysts with or without fibrosis), increase in blood vessels [graded as 0 (normal) or 1 (increased)]), presence or absence of new cartilage within the bone, thickened trabeculae with evidence of thickening determined as greater than 5 µm in at least 3 regions of subjects sections [graded as 0 (normal) or 1 (increased)], breaching of the tidemark integrity between the cartilage and bone interface, and inflammation, defined as fibrovascular infiltration of marrow with at least 2 regions per section demonstrating an increased number of macrophages and osteoclasts (>10 per section). Based on this system a summed score ranging from 0- 7 was reached, with 0 being normal and 7 the highest damage, as summarised in Figure 1.

Immunohistochemistry analysis of BML tissue was also assessed for the presence of nerve staining by using a PGP9.5 primary anti-human antibody, with a donkey anti-mouse conjugated horseradish peroxidase secondary antibody development system. Tissue sections were deparaffinised, rehydrated and heated in citrate buffer (10 mM, pH 6.0) for 1 hour at 70 degrees. Sections were then simultaneously permeabilised and blocked in phosphate-buffered saline (PBS) containing 1.0% bovine serum albumin (BSA), 0.4% Triton X-100 and 5% normal donkey serum for 1.5h at room temperature.

Prior to primary antibody incubation, the endogenous peroxidase activity was blocked with 1% H_2_O_2._ Nerve tissue was analysed as previously described in joint tissue by PGP 9.5 staining (11). For the presence of nerve staining, slides were then incubated overnight at room temperature with a primary anti-PGP9.5 mouse monoclonal antibody diluted in 1% BSA (dilution 1:200) (Abcam–ab8189) and subsequently with HRP-conjugated donkey anti-Mouse IgG secondary antibody (dilution 1:400) at room temperature (RT) for 2 hours. (A24500 Thermo Fisher). All slides were developed with 3,3′-diaminobenzidine (DAB) solution (Abcam ab64238) at room temperature for 2 minutes and Counterstained with Harris haematoxylin.

*Definitions of bone marrow lesions and cysts*

Bone marrow lesions are characterised by magnetic resonance imaging as diffuse areas of high-intensity signal on T2-weighted, fat-saturated MRI or in short tau inversion recovery sequences.

Cysts are fluid filled structures apparent on MRI and may also be identified histologically within subchondral bone regions corresponding to MRI-localised in BMLs, as areas of infiltration by inflammatory cells and vascular proliferation, surrounded by regions of fibrosis.

*Blood vessels density quantification*

For assessment of blood vessels, the number of blood vessels per group was counted within the standardized depth and width of the tissue, 7.2mm and 15.5 mm respectively. Sections were of standard thickness, and therefore BV numbers given here therefore represent densities per unit total tissue volume. The maximum number of blood vessels in the control group was counted in all tissue sections and an average was taken, accounting for normality. The mean BV density in the normal group was 12 and a cut-off point was set at 15. Blood vessel density higher than 15 was suggestive of hypervascularity.

*Inter-observer reliability assessment between scorers*

Inter-observer reliability was assessed in Groups 1 and 2 during development, when 2 observers (SK, NS) scored independently of each other where NS was blinded to BML status and diagnosis. Inter-observer reliability was also separately assessed by 2 observers (SK, MS) in group 3 where BML status was unknown
