# Supplementary material for: The osteoarthritis bone score (OABS): a new histological scoring system for the characterisation of bone marrow lesions in osteoarthritis
Source: Osteoarthritis Cartilage. 2022 May;30(5):746–55. doi: 10.1016/j.joca.2022.01.008 (PMC9395274; doi:10.1016/j.joca.2022.01.008)
Supplement: Multimedia component 2 [file mmc2.docx]

**Supplementary Data**

**Supplementary Table 1. Osteoarthritis Bone Score Rasch analysis individual item fit statistics**

| Item | Item descriptor | Location | Standard Error | Fit Residual | Chi^2^ | Probability |
| --- | --- | --- | --- | --- | --- | --- |
| 1 | Cysts | 2.211 | 0.202 | -0.399 | 1.562 | 0.458 |
| 2 | Fibrosis | 0.094 | 0.187 | -1.663 | 14.351 | 0.001 |
| 3 | Blood vessels | -0.499 | 0.202 | 1.291 | 4.763 | 0.092 |
| 4 | Cartilage | 0.825 | 0.180 | 0.121 | 1.966 | 0.374 |
| 5 | Trabeculae thickened | -1.575 | 0.253 | -0.255 | 8.892 | 0.012 |
| 6 | Tidemark integrity | -1.037 | 0.223 | -0.166 | 0.409 | 0.815 |
| 7 | Inflammation | -0.010 | 0.189 | -2.061 | 10.587 | 0.005 |

**Item descriptor key:** Fibrosis; fibrotic connective tissue within bone marrow space, Blood vessels; number of blood vessels within the subchondral region of interest, Cartilage islands; new cartilage within bone, Inflammation; cellular infiltrates.

**Supplementary Table 2. Osteoarthritis Bone Score Rasch analysis overall item and person fit statistics**

|  | Item | | Person | |
| --- | --- | --- | --- | --- |
|  | Location | Fit residual | Location | Fit residual |
| Mean | 0.000 | -0.445 | 1.196 | -0.225 |
| SD | 1.250 | 1.121 | 1.430 | 0.679 |
| Skewness | 0.742 | -0.054 | -0.401 | 0.765 |
| Kurtosis | 0.655 | -1.355 | -0.620 | 0.058 |
| N | 7 | | 203 | |

**Supplementary Table 3: Osteoarthritis Bone Score polychoric correlation matrix**


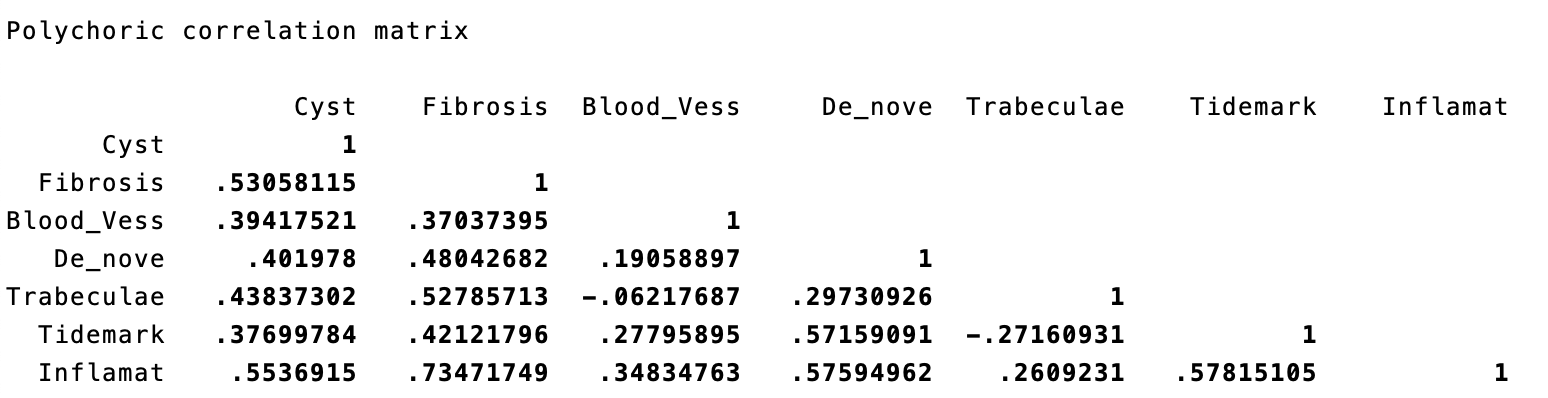


**Item descriptor key:** Fibrosis; fibrotic connective tissue within bone marrow space, Blood vess; number of blood vessels within the subchondral region of interest, De-nove; cartilage islands (new cartilage within bone), Trabeculae; trabeculae thickened, Tidemark: tidemark integrity, Inflammat; cellular infiltrates.

**Supplementary Table 4: Osteoarthritis Bone Score factor analysis**


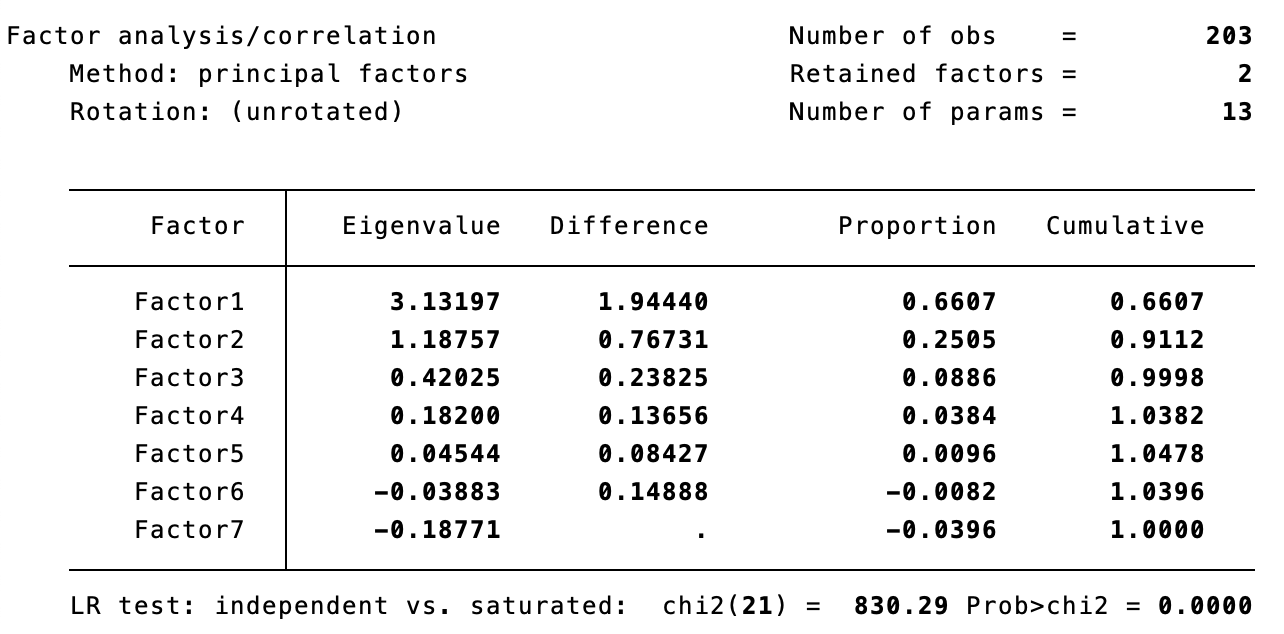


**Supplementary Table 5: Osteoarthritis Bone Score rotated factor loadings (pattern matrix) and unique variances sorted**


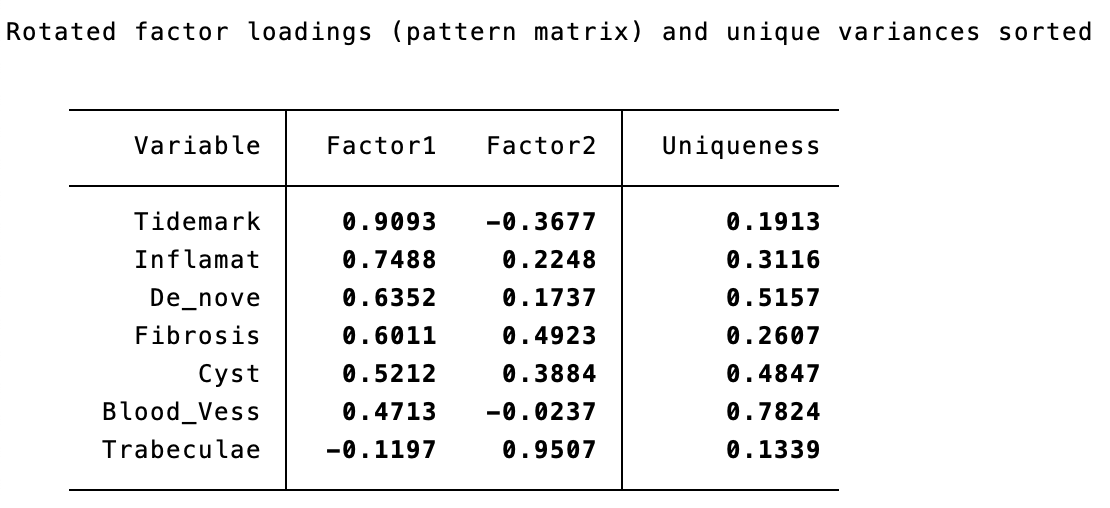


**Item descriptor key:** Fibrosis; fibrotic connective tissue within bone marrow space, Blood vess; number of blood vessels within the subchondral region of interest, De-nove; Cartilage islands, new cartilage within bone, Trabeculae; trabeculae thickened, Tidemark: tidemark integrity, Inflammat; cellular infiltrates.

**Supplementary Table 6: Osteoarthritis Bone Score factor rotation matrix**


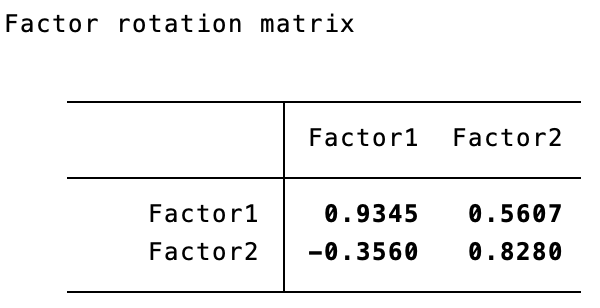


**Inter-observer reliability results between independent scorers**

ICC for inter-observer reliability (SK, NS) for OABS within Groups 1 and 2 of the development set was 0.95 (0.88 to 0.97), p<0.001.

ICC for inter-observer reliability (SK, MS) for OABS within Group 3 of the development set was 0.81 (0.38 to 0.94), p<0.001

**Supplementary Table 7: Individual case frequency data for MOAKS scores and OABS scores for each BML sample in the developmental set (Group 1)**

| **Eff/Syn_MOAKS** | **0** | **1** | **2** | **3** |
| --- | --- | --- | --- | --- |
| **PA044** | 0 | 1 | 0 | 0 |
| **PA069** | 0 | 1 | 0 | 0 |
| **PA081** | 0 | 1 | 0 | 0 |
| **PA083** | 0 | 0 | 1 | 0 |
| **PA116** | 0 | 0 | 1 | 0 |
| **PA123** | 0 | 0 | 1 | 0 |
| **PA124** | 0 | 1 | 0 | 0 |
| **PA125** | 0 | 1 | 0 | 0 |
| **PA126** | 0 | 1 | 0 | 0 |
| **PA129** | 0 | 0 | 1 | 0 |
| **Mean** | 0 | 0.6 | 0.4 | 0 |
| **%** | 0 | 60 | 40 | 0 |

| **BML_MOAKS** | **0** | **1** | **2** | **3** | **Cartilage_MOAKS** | **0** | **1** | **2** | **3** |
| --- | --- | --- | --- | --- | --- | --- | --- | --- | --- |
| **PA044** | 6 | 5 | 3 | 1 | **PA044** | 0 | 5 | 4 | 5 |
| **PA069** | 4 | 6 | 3 | 2 | **PA069** | 2 | 1 | 3 | 8 |
| **PA081** | 9 | 2 | 2 | 2 | **PA081** | 3 | 0 | 3 | 8 |
| **PA083** | 12 | 2 | 1 | 0 | **PA083** | 4 | 1 | 3 | 6 |
| **PA116** | 8 | 6 | 1 | 0 | **PA116** | 1 | 0 | 4 | 9 |
| **PA123** | 1 | 10 | 4 | 0 | **PA123** | 0 | 0 | 3 | 11 |
| **PA124** | 8 | 5 | 1 | 1 | **PA124** | 0 | 1 | 6 | 7 |
| **PA125** | 10 | 3 | 2 | 0 | **PA125** | 8 | 1 | 2 | 3 |
| **PA126** | 11 | 4 | 0 | 0 | **PA126** | 0 | 2 | 5 | 7 |
| **PA129** | 8 | 3 | 2 | 2 | **PA129** | 1 | 3 | 5 | 5 |
| **Mean** | 7.7 | 4.6 | 1.9 | 0.8 | **Mean** | 1.9 | 1.4 | 3.8 | 6.9 |
| **%** | 51.333 | 30.667 | 12.667 | 5.3333 | **%** | 13.571 | 10 | 27.143 | 49.286 |

| **OABS** | **0** | **1** | **2** | **3** | **4** | **5** | **6** | **7** |
| --- | --- | --- | --- | --- | --- | --- | --- | --- |
| **PA044** | 0 | 1 | 1 | 1 | 1 | 1 | 1 | 1 |
| **PA069** | 0 | 1 | 1 | 1 | 1 | 1 | 1 | 1 |
| **PA081** | 1 | 1 | 1 | 1 | 0 | 1 | 1 | 1 |
| **PA083** | 5 | 0 | 0 | 1 | 0 | 1 | 0 | 0 |
| **PA116** | 0 | 1 | 1 | 1 | 1 | 1 | 1 | 1 |
| **PA123** | 0 | 1 | 1 | 1 | 1 | 1 | 1 | 1 |
| **PA124** | 0 | 1 | 1 | 1 | 1 | 1 | 1 | 1 |
| **PA125** | 1 | 1 | 1 | 1 | 0 | 1 | 1 | 1 |
| **PA126** | 1 | 1 | 1 | 1 | 0 | 1 | 1 | 1 |
| **PA129** | 0 | 1 | 1 | 1 | 1 | 1 | 1 | 1 |
| **Mean** | 0.8 | 0.9 | 0.9 | 1 | 0.6 | 1 | 0.9 | 0.9 |
| **%** | 11.429 | 12.857 | 12.857 | 14.286 | 8.571 | 14.286 | 12.857 | 12.857 |

Table legend: the columns on the left represent participants’ ID and the numbers 0-3 correspond to MOAKS scores for each sample. The number 0-7 correspond to OABS scores for each sample.

Abbreviations: Effusion/Synovitis (Eff/Syn), Bone marrow lesions (BML), Osteoarthritis Bone Score (OABS), MRI Osteoarthritis Knee Score (MOAKS)
